# Some Limitations of Public Sequence Data for Phylogenetic Inference (in Plants)

**DOI:** 10.1371/journal.pone.0098986

**Published:** 2014-07-07

**Authors:** Cody E. Hinchliff, Stephen Andrew Smith

**Affiliations:** Department of Ecology and Evolutionary Biology, University of Michigan. Ann Arbor, Michigan, United States of America; Montreal Botanical Garden, Canada

## Abstract

The GenBank database contains essentially all of the nucleotide sequence data generated for published molecular systematic studies, but for the majority of taxa these data remain sparse. GenBank has value for phylogenetic methods that leverage data–mining and rapidly improving computational methods, but the limits imposed by the sparse structure of the data are not well understood. Here we present a tree representing 13,093 land plant genera—an estimated 80% of extant plant diversity—to illustrate the potential of public sequence data for broad phylogenetic inference in plants, and we explore the limits to inference imposed by the structure of these data using theoretical foundations from phylogenetic data decisiveness. We find that despite very high levels of missing data (over 96%), the present data retain the potential to inform over 86.3% of all possible phylogenetic relationships. Most of these relationships, however, are informed by small amounts of data—approximately half are informed by fewer than four loci, and more than 99% are informed by fewer than fifteen. We also apply an information theoretic measure of branch support to assess the strength of phylogenetic signal in the data, revealing many poorly supported branches concentrated near the tips of the tree, where data are sparse and the limiting effects of this sparseness are stronger. We argue that limits to phylogenetic inference and signal imposed by low data coverage may pose significant challenges for comprehensive phylogenetic inference at the species level. Computational requirements provide additional limits for large reconstructions, but these may be overcome by methodological advances, whereas insufficient data coverage can only be remedied by additional sampling effort. We conclude that public databases have exceptional value for modern systematics and evolutionary biology, and that a continued emphasis on expanding taxonomic and genomic coverage will play a critical role in developing these resources to their full potential.

## Introduction

The GenBank nucleotide database [1] contains more than one hundred million sequences representing more than 275,000 species of life. The successful use of these data to reconstruct comprehensive phylogenies for many large clades has illustrated their potential for phylogenetic inquiry [2], [3], with the implication that this potential may extend to the broadest scales, e.g. the tree of life itself [3], [4]. By some estimates however, GenBank contains samples of only 3% of Earth's species [5], and studies using sequence data mined from public databases have demonstrated enigmatic results [6]–[8]. Public sequence data have clear potential for evolutionary biology and hypothesis-testing at very broad scales, but their structure can have significant implications regarding the limits of phylogenetic inference [9], [10]. The extent and severity of these limits for existing resources such as GenBank remains largely unexplored (but see [3], [4]). Here we demonstrate the potential and the limits to inference of the NCBI GenBank database for comprehensive phylogenetic studies using the land plants, a monophyletic, ancient, and very biodiverse group with over 300,000 extant species [11] as an example.

## Results and Discussion

### Leveraging public sequence data

**Figure 1:**
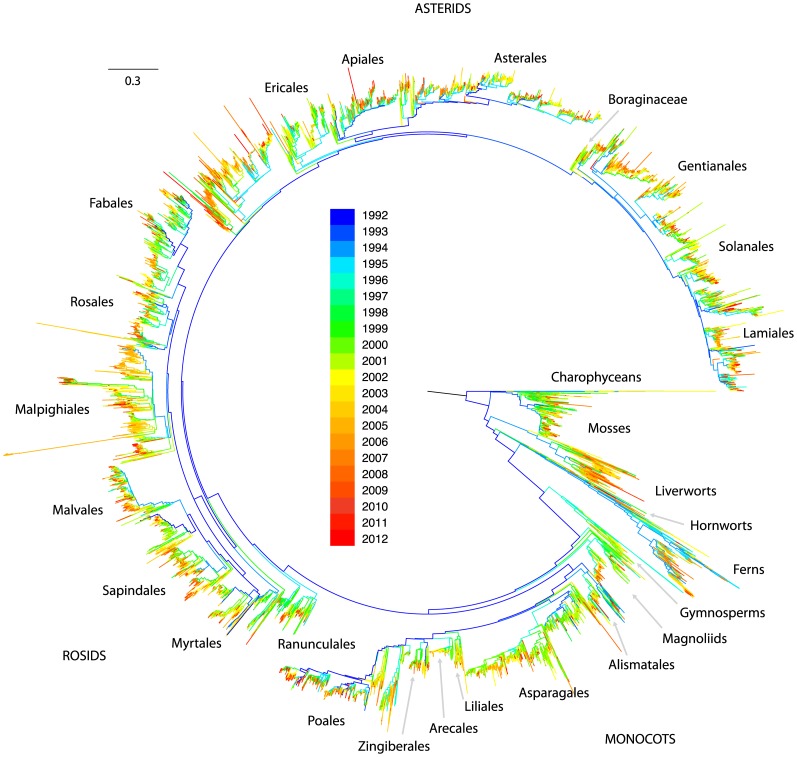
Phylogeny of land plants generated from nucleotide sequence data in GenBank release 185. Tips represent extant genera and branch lengths correspond to substitutions per site. Each branch is labeled according to the age of the oldest exemplar sequence in GenBank for any of its descendant tips, with blue branches representing lineages with older exemplar sequences and red branches showing lineages that have only recently been added. The total number of genera represented in the tree is 13,093. A large version of this figure with legible tip names is presented in File S1.

We compiled nucleotide sequence data from GenBank for the land plants, including the closely related Charophycean algae [12]–[14] to facilitate rooting. A total of 128 markers were selected for their relatively broad phylogenetic coverage, including 109 chloroplast, 14 mitochondrial, and 5 nuclear markers including the nuclear ribosomal internal and external transcribed spacers (see Table S1 for a complete list). We chose not to include additional nuclear markers because of challenges of homology assessment at deep phylogenetic scales. We used the program PHLAWD [7] to gather data from GenBank release 185 for these 128 markers, resulting in a data set including 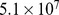 genetic sequences and representing over 100,000 plant and algal species. To maximize coverage and reduce computational complexity, we summarized the available sequence data for all of the 13,093 genera for which at least one sequence was available. For each of these genera, we selected the longest available sequence at each of our target loci that had been sequenced for any taxonomic child of that genus, which produced a set of sequence data (usually from multiple species) that was used to represent that genus in the alignment. Maximum likelihood [15] was used to infer phylogeny using this alignment, yielding the tree topology presented in Fig. 1.

**Figure 2:**
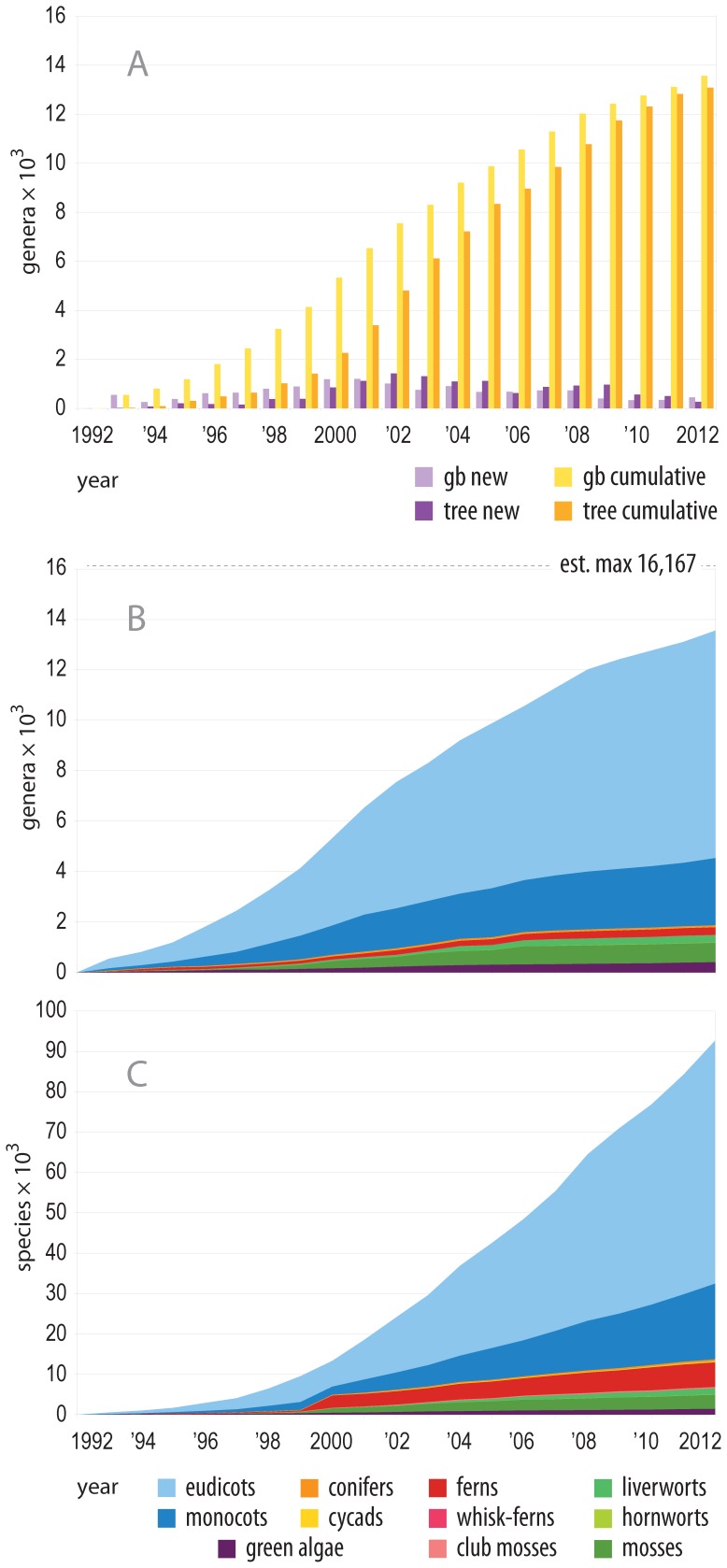
Taxon sampling through time on GenBank and the ages of exemplar sequences. A: purple bars indicate the number of new generic exemplars that were: added each year to GenBank (light purple); and the number of exemplar sequences from that year that were used in the phylogeny presented in Figs. 2 and 5 (dark purple). Yellow bars indicate cumulate values. The lag in the total number of genera represented in the phylogeny is due to the fact that the sequence selection procedures prefer the longest available exemplar sequence, which is often not the oldest. B and C: cumulative numbers of genera (B) and species (C) represented by sequences on GenBank, with colors labeling major groups. Species representation continues to grow at a relatively constant rate, but the rate of new genus addition is slowing.

Despite the vast amount of data available on GenBank, only about one third of recognized plant species were represented in GenBank release 185. At deeper taxonomic levels this coverage is considerably better, with about 83% (about 13,400 of 16,167) of recognized land plant genera [16] represented by at least one sequence. The rate of species accumulation on GenBank has stayed relatively constant since the mid 1990's and shows no signs of reaching saturation (Fig. 2, C). Coverage at the generic level however, is approaching the estimated maximum of 16,167 (Fig. 2, B), representing a landmark achievement for plant systematists. Our ability to reconstruct the phylogeny of extant plants has grown as a function of this increase in lineage representation through time (Fig. 1). Early, often broadly inclusive studies [17]–[21] resolved many deep divergences (blue branches of Fig. 1), while myriad more detailed studies [22]–[30] for example) have contributed to resolution near the tips and also increased confidence in deep relationships.

The comprehensive phylogenies made possible by such achievements facilitate inquiry into questions of broad interest to the scientific community. For example, we used the tree topology presented in Fig. 1 to assess the level of monophyly in the land plant classification (as described in the Materials and Methods), finding that monophyly cannot be rejected for 75.6% of the 680 families of Embryophyta in use in the Genbank taxonomy at the time of writing. To facilitate additional inquiry into more detailed questions, we also provide a time calibrated version of the Fig. 1 phylogeny using node age constraints from a recent dating analysis of angiosperms [31] in the supplemental materials.

### The impact of missing data

It is well-understood that high proportions of missing data (or more specifically, that the distribution and nature of such missing data) can impact phylogenetic inference [3], [6], [32]. Most large alignments with broad phylogenetic scope compiled from public sequence databases have relatively high proportions of missing data [3], [6]–[8]; for instance, the alignment used to generate the tree of Fig. 1 (13,093 taxa 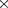 128 loci) contained 96.4% missing data. To measure the limiting effect of these absent data, we used the partial decisiveness metric 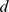 [10]. This metric indicates the proportion of all possible edges across all possible trees that are distinguishable given the available data (that is, given the distribution of missing data). 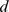 is distributed on interval 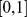 with 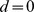 indicating a fully uninformative alignment in which no edges may be informed because the available data are not sampled densely enough to permit phylogenetically informative comparisons, and 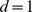, indicating that all possible edges may be informed (i.e. all possible trees may be inferred). We note that edge *distinguishability*, which is measured by *d*, is not synonymous with edge *support* (we assess branch support with other measures; see corresponding sections), and that 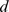 does not measure phylogenetic signal. An edge is considered distinguishable—that is, the data are decisive for that edge—if the taxonomic sampling is such that the edge *may* be informed by at least some of the present data, but this does not imply that those data *will* support a topology containing that edge [9], [10].

The partial decisiveness of our comprehensive alignment was estimated to be 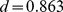, indicating that GenBank's sampling is sufficient to inform all but 13.7% of the potential phylogenetic relationships among represented plant genera. This statistic represents a fairly conservative estimate of GenBank's phylogenetic utility in the sense that while 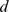 considers all possible edges, only a small subset of those are likely to occur in phylogenetic trees. Accurate phylogenies may be reconstructed while 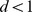 as long as the data are decisive for the edges present in those tree(s) that actually represent the phylogenetic history of the sampled organisms [6].

**Figure 3:**
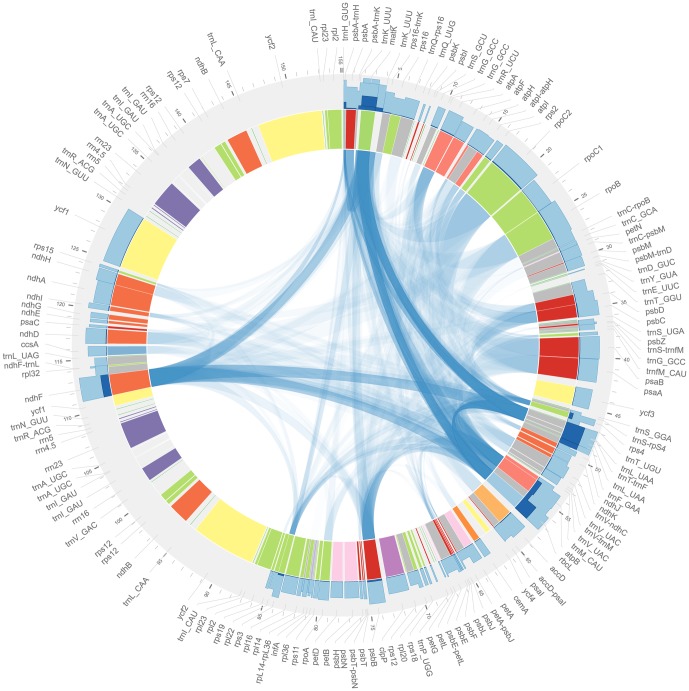
Taxonomic sampling depth and overlap for chloroplast loci in GenBank. Data shown are representation of genera in GenBank release 185 for all chloroplast loci sampled for this study, superimposed on a genome map of the *Coffea arabica* genome. Overall generic sampling depth across the chloroplast is quite low. Dark blue bar plots on the outside of the ring show the number of genera 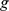 represented by at least one exemplar sequence for each locus, out of 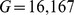 total genera, while light blue bars show 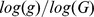, and illustrate relative sampling proportions among loci when absolute proportions are small. Ribbon plots in the center of the figure identify pairs of loci, and indicate the proportion of genera that are represented by exemplar sequences for both loci in the pair. Dark ribbons label locus pairs that are co-represented for many genera; light ribbons label pairs that are co-represented for few. Locus colors correspond to gene groupings by function, and tick marks show linear distance in kilobases. The most well sampled locus in our entire alignment was *trnT–trnL–trnF*, with 55% of genera represented. Mitochondrial and nuclear loci are not shown in the figure, but the most well-sampled nuclear markers were ITS (53%) and ETS (8%; similar to *rps4* in the figure), and for the mitochondrion *atpA* (7%), *rps3* (5%), *matR* (5%), and *atp1* (5%); all other nuclear and mitochondrial markers were sampled for fewer than 4% of genera. Exact counts of genera represented for all markers sampled in this study are available in Table S3.

**Figure 4:**
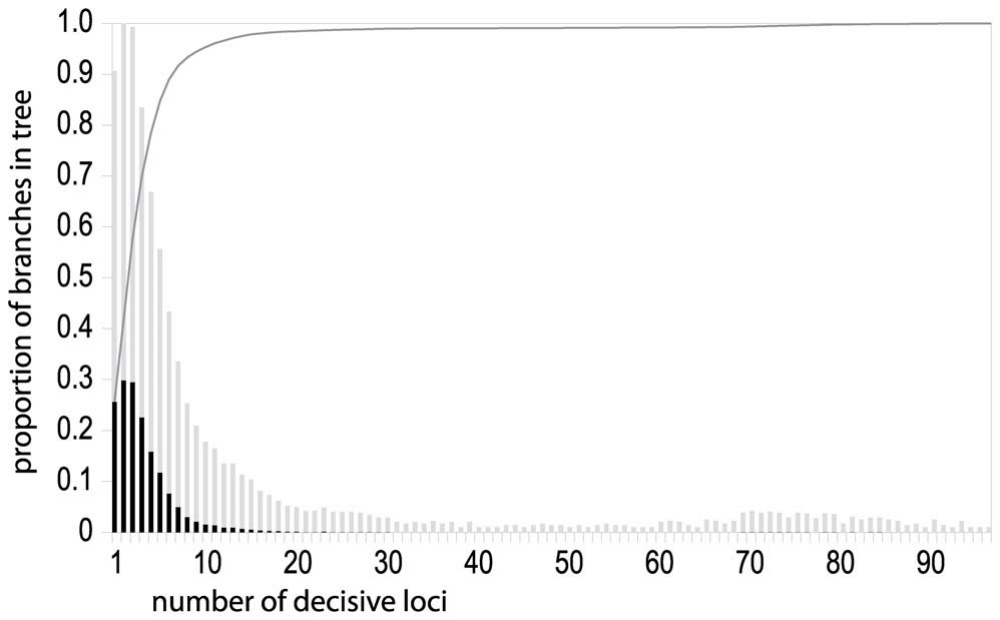
Genetic sampling depth for branches in the ML generic topology of Figs. 1, 5, and 6. Data shown are the proportion of branches for which each number of loci have decisive taxon sampling. Black bars are actual proportions, and grey bars are square roots of each proportion, which illustrate relative differences when proportions are very small. The dark grey line is the cumulative proportion of branches, which indicates the proportion of branches in the tree for which the number of loci with decisive taxon sampling is less than or equal to the indicated 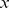 value. Exact values used to create this figure are presented in Table S2.

Phylogenetic inference relies on the co-sampling of homologous data across lineages; high levels of lineage representation allow the distinguishability of many more edges than low levels simply because lineages for which no data are present can only be arbitrarily placed. Ideally, phylogenetic datasets should contain an adequate number of informative loci (we usually assume that more is better), each sampled for many lineages, thus allowing many edges to be informed by relatively large amounts of data. In Fig. 3, we present patterns of lineage and locus sampling depth across the entire plant chloroplast genome, which comprises the great majority of phylogenetically informative sequence data for plants. In general, chloroplast loci show highly asymmetrical lineage sampling (indicated by dark blue bar plots in the outer ring), and the frequency at which pairs of loci have been sampled for the same lineages is in quite low overall (indicated by the blue ribbons connecting pairs of loci). Only a handful of loci—*atpB*, *matK*, *ndhF*, *psbA-trnH*, *rbcL*, *rps4*, and *trnT-trnL-trnF*—show relatively high levels of lineage representation or taxonomic overlap (primarily with one another). Similar patterns hold for loci in the mitochondrial and nuclear genomes (data not shown), and the problem of sparse data coverage is in fact exacerbated in the case of nuclear genes by challenges associated with homology assessment at deep phylogenetic scales. Only the nuclear ribosomal internal transcribed spacer (ITS) shows levels of lineage sampling similar to the heavily sampled chloroplast loci named above (Fig. 3; Table S3). The partial decisiveness (*d*) of GenBank's nucleotide data, and the great majority of our information about phylogenetic relationships among plants, comes from these fewer than 10 loci (Figs. 3, 4). Many of these best sampled loci, however, are relatively fast evolving (especially ITS and chloroplast intergenic spacers), and contain relatively little phylogenetic signal for resolving deep branches in the tree.

**Figure 5:**
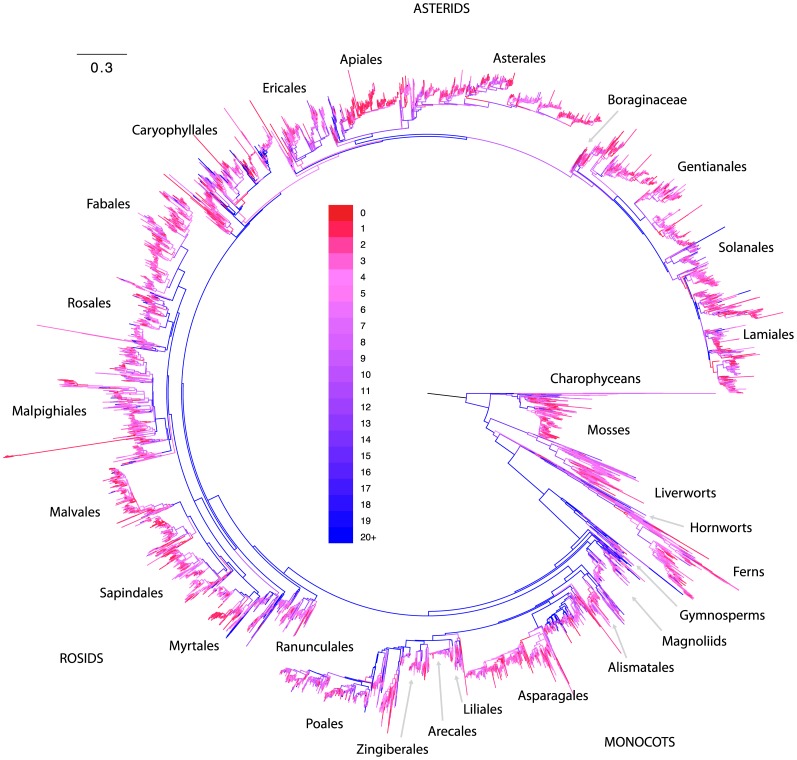
Generic phylogeny of plants with branches colored according to the number of loci with decisive taxon sampling. Branches for which large numbers of loci have phylogenetically decisive taxon sampling are blue, while branches with low numbers are pink to red. Blue branches have a higher capacity than red branches to be informed by the data. Most branches in the tree are able to be informed by data at relatively few loci, but deep branches are generally able to be informed by many. A large version of this figure with legible tip names is presented in File S2.

We assessed the extent and potential impact of this sparse data coverage at a more targeted scale by calculating the number of loci in the alignment which had phylogenetically decisive taxon coverage for each branch in the ML tree (see Materials and Methods). These branch-specific patterns of data decisiveness across the land plant genus phylogeny are presented in Fig. 5, and additional figures identifying the individual branches capable of being informed by each locus are presented in the file titled “Supplemental tree figures” that is available in the Data Dryad repository associated with this article. Very deep branches in the tree are informed by many loci (blue hues in Fig. 5), but branches near the tips are informed by relatively few (red hues in Fig. 5). Figure 4 plots sampling depth (in this case the number of loci with decisive taxon sampling) by the proportion of branches in the tree, and demonstrates that the great majority of branches in the tree are informed by relatively few loci.

The patterns shown in Figs. 4 and 5 suggest that GenBank data may lack the sampling depth required to accurately infer comprehensive plant phylogenies. We combined data at the generic level to maximize genetic coverage for the resulting tips, but even this did not entirely overcome sampling issues at shallow phylogenetic depths. This is a significant limitation for a variety of evolutionary analyses that depend on trees—shallow to mid-depth regions of trees encode a far greater amount of phylogenetic information (by sheer number of branches and nodes alone) than deep branches, and have a very high capacity to inform a broad variety of questions regarding evolutionary processes in plants. Thus, we argue that the accurate inference of shallow relationships is critical for many important questions in evolutionary biology (e.g. any analysis involving lineage diversification). At shallower taxonomic depths than genera, however, the limiting effects of sparse data coverage are even stronger than those we present here. Overcoming these limits will require increased sampling to improve both the genetic depth as well as the taxonomic coverage of the data.

### Measuring branch support

Estimating branch support is an important mechanism of assessing confidence in topology, but doing so with large, sparse matrices can be challenging. Traditional measures such as bootstraps do not perform well with very high proportions of missing data [6] and replicate tree searches (e.g. Bayesian MCMC, standard bootstrapping, and jackknife) on large alignments can be prohibitively time consuming. In the case of our alignment, a single ML tree search took several weeks using 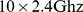 Intel Xeon processor cores and more than 40 GB of memory; running thousands or more of these is not feasible. We therefore implemented a measure of branch support that is relatively fast to calculate given a single tree and an alignment (even for large alignments), which relies on the information criterion (IC) framework presented by [33]. Specifically, we used the ICA statistic (defined in that paper), which is a branchwise measure of support based on information theory that quantifies, for a given bipartition (e.g. the one implied by a given branch in a tree), the level of congruence or conflict (with that bipartition) across a set of topologies. The ICA score varies on the interval [−1,1], with 1 indicating perfectly congruent supporting information—the specified bipartition is observed in all of the topologies; −1 indicating perfectly congruent conflicting information—the specified bipartition is never observed, but rather a single conflicting bipartition is observed in all the topologies; and 0 indicating perfectly equivocal information—all observed bipartitions occur at equal frequency. A positive ICA score in general indicates that the specified bipartition is observed at a higher frequency than any single alternative (i.e. conflicting) bipartition, whereas a negative ICA score indicates that some other alternative bipartition occurs at a higher frequency.

**Figure 6:**
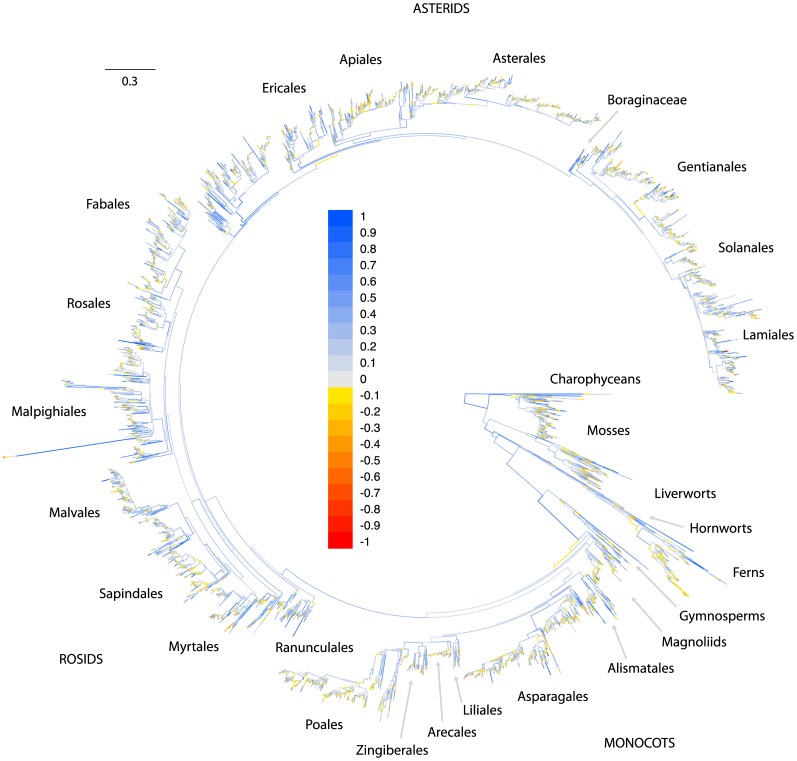
Generic phylogeny of plants with branches colored according to ICA support values. Branches with strong positive values (high support) are light blue, while branches with low positive values (low support) are gray, and branches with negative values (which imply relatively strong conflicting signal in the data) are colored yellow to red. Most branches deep in the tree are moderately well supported, whereas most strongly supported branches occur within smaller clades, and most branches that appear to be in conflict with the signal from the alignment occur near the tips of the tree. Terminal branches (i.e. tips) were pruned from the tree for display purposes, as they do not have meaningful support values. A large version of this figure with legible tip names is presented in File S3.

Since computational limits prevented us from generating topology replicates for the entire tree, we generated replicates using an approach that we call a localized taxon quartet jackknife, which consisted of selecting tips at random from clades defined by the complete ML tree, and inferring topology for these randomized tip subsamples to generate topology replicates. Each replicate contained a quartet of tips selected to guarantee that any topology inferred for those tips would either be consistent with a given targeted branch in the original ML topology, or would conflict with that branch (see Materials and Methods for a more complete explanation of the subsampling procedure). For each branch in the ML tree, 500 representative ML topologies were generated using these randomly selected quartet replicates, and the resulting topology set was used to calculate the ICA score for that branch. We present this information in Fig. 6, which contains the ML topology colored according to the ICA score estimated for each branch. Blue branches are those with positive ICA scores, that is, the quartet topology consistent with those branches was observed more often than either of the possible conflicting quartet topologies. Branches with negative scores are colored yellow to red, and indicate branches that are not supported by the data—the most frequently resolved quartet topology in these cases was in conflict with the branch. These poorly supported (or controversially resolved) branches are primarily concentrated near the tips of the tree, where data coverage, and by extension the number of loci with decisive taxon coverage, for each branch are low. It is also likely that better resolutions for some of these controversial branches may have been found by running the ML optimization procedure for longer, but such an exhaustive ML search was not feasible, as is often the case for datasets of this size.

**Figure 7:**
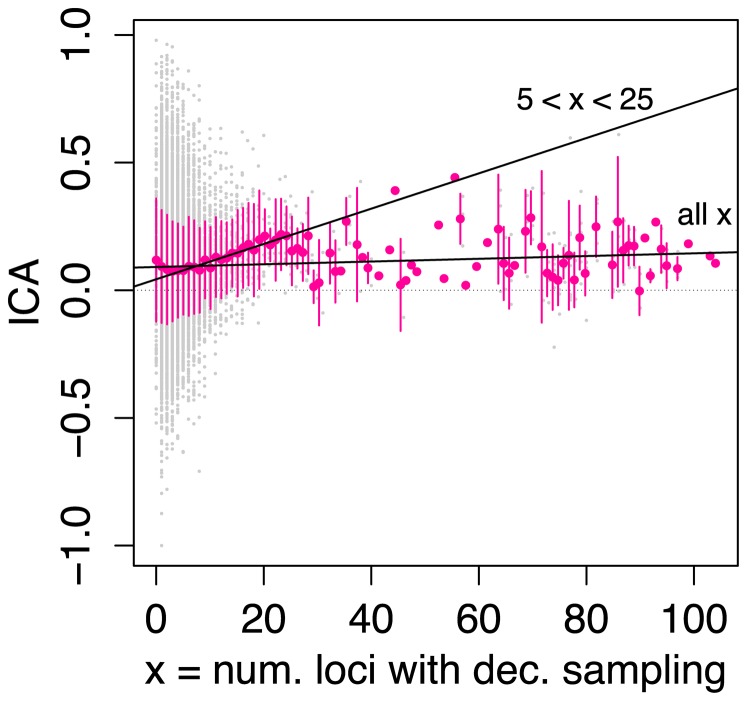
Correlation of branch support (ICA) with locus sampling depth (number of loci with decisive taxon sampling). Original data points correspond to individual internal branches in the ML topology, and are shown in gray. Large colored dots represent mean ICA values for all branches with the corresponding number of loci with decisive sampling, and error bars extend to plus or minus the standard deviation of the data from each mean. Two regression lines are plotted, one for the entire dataset (labeled “all 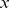
”; 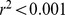, 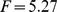, 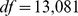, 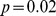) and one for only those branches with greater than five but fewer than 25 loci with decisive sampling (labeled “
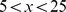
”; 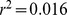, 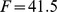, 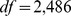, 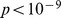).

To more specifically address the question of whether increased sampling depth (measured here as the number of decisive loci) affects confidence in branch reconstruction, we used a simple linear regression to assess the correlation between the number of decisive loci for a branch, and its ICA score (Fig. 7). Very strong branch support values (i.e. ICA close to 1) are elusive in this dataset, even with high numbers of decisive loci, and the relationship between ICA score and the number decisive loci is correspondingly weak when the entire dataset is analyzed (the “all 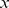
” regression line in Fig. 7; 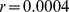, 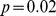). However, very few branches in the tree are informed by more than 25 loci, and the combination of this sparse sampling with the high variance in these data places strong limits on our ability to infer patterns at this scale. It is likely that at least some of the variance in branch support is due to “dirty data” in GenBank, such as misidentified taxa or poor quality sequences, which lead to spurious topology inference when those data are subsampled. Another process that may affect support for deep branches is that even though many loci in the alignment contain decisive taxon sampling for these branches, individual randomized representative taxon quartet replicates for deep branches may often not subsample quartets with decisive sampling, simply because the number of possible quartet combinations for deep branches is large. Near the tips, there is a stronger tendency for subsampled taxa to be represented for the same loci, thus potentially increasing the level of phylogenetic signal in mid-depth replicates. Future studies to more thoroughly characterize the behavior of the ICA statistic, as well as the localized taxon quartet jackknife we present here, would be valuable.

To circumvent the limits imposed by the sparse sampling of branches with high numbers of decisive loci, we performed an additional regression which we limited to a subset of the data. The mean ICA values for branches with relatively low numbers of decisive loci (i.e. fewer than about 25) suggest a positive correlation, though branches informed by very few loci (i.e. fewer than about 5) show a decreasing trend in ICA compared to those with more. We conjecture that the decreasing support values for branches with fewer than about 5 decisive loci may be due to increasing levels of conflicting signal as more data are added, up to the point where the dominant signal becomes strong enough to reverse the trend (other plausible explanations certainly exist). We therefore limited the second regression to branches with greater than 5 but fewer than 25 decisive loci (the “
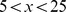
” line in Fig. 7), which we propose constitutes a representative and sufficiently densely sampled subset of the data to accurately quantify any existing trend. In these subsampled data, we find a positive correlation between branch support and the number of loci capable of informing the branch. This relationship is strongly supported (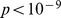), but even so, the correlation itself is weak (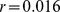), suggesting that even with relatively high levels of data coverage (in this case, up to 25 loci, which is higher than many phylogenetic studies being published today), confident inference of at least some phylogenetic relationships may remain challenging. This observation is consistent with studies using very large and densely sampled alignments, which have nonetheless yielded trees containing numerous poorly supported branches [6], [13], [23], [34]. The reasons for this are unclear, but may be related to conflicting phylogenetic signal or in some cases to an overall lack of informative sites even at genome-wide scales.

### Concluding remarks

The utility of public sequence databases for phylogenetic inference has reached never before seen levels, facilitating the inference of phylogeny at both broad and deep scales for major groups in the tree of life (Figs. 1 and 2). In plants, we are nearing a threshold where nearly every known land plant genus is represented in GenBank by at least one exemplar sequence, making phylogeny inference across all lineages of land plants possible at relatively fine scales (Figs. 2 and 5). These comprehensive phylogenies have already shown a unique potential to address broad evolutionary questions that may be difficult to test at less inclusive scales [6], [8], [32], [35], [36], and many opportunities exist for researchers who wish to exploit the potential of public sequence databases. Nevertheless, lineage representation remains extremely low for all but a handful of genetic markers (Fig. 3), and resources such as GenBank remain heavily limited by this (Figs. 4, 5, and 6).

The most obvious solution to the problem of low data coverage is simply to increase sampling for informative loci for lineages without it, and indeed one of the implications of the work we present here is that the accurate resolution of comprehensive land plant phylogenies may require the collection of a significant amount of additional sequence data (Figs. 5, 6). This implication is corroborated by the results of previous work by Sanderson [4], which showed that phylogenetically informative sampling is very low for a large number of eukaryotic lineages. In fact, that study found that land plants were among the best sampled major lineages of eukayotic life (after vertebrates), clearly illustrating that despite the relative low sampling depth for land plant genera (Figs 3, 5), the situation is even more extreme in almost all other parts of the tree of life. We suggest that one proactive response to this situation would be to continue to fund and pursue opportunities to improve both taxonomic as well as genetic coverage across the tree of life.

Statistics such as *d*, ICA, and the measures we have implemented here using these theoretical foundations (Figs. 5 and 6) provide useful tools that can allow the rapid and accurate identification of potential problem areas even in very large phylogenies, and thus may enable efficient and cost-effective collection of targeted data to improve taxonomic and genetic coverage. The concept of data decisiveness in general can be expected to remain a useful theoretical background for data-mining approaches across diverse lineages on the tree of life, and statistics such as 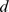 as well as related methods [37], [38] may be able to provide a more nuanced assessment of the phylogenetic potential of large databases than previous approaches have allowed [3], [4].

The continued accrual of novel sequence data and improvements to taxonomic coverage at the species level will be required to facilitate broad application of these resources at fine evolutionary scales. Improvements to coverage, however, will lead to increasingly larger datasets, which can pose significant technical challenges for analysis methods. An alignment of 100,000 plant species sampled for the same 128 genetic loci as this study resulted in an alignment text file over 6 GB in size, which exceeded the capabilities of available phylogenetic search software. If the relatively stable rate of species accumulation in GenBank remains so for the next four decades, we will reach the estimated (minimum) land plant species diversity of 300,000 around the year 2044, but technical challenges related to computational tractability (given currently available software) would prevent reconstruction of trees with 300,000 plant species, just as those problems prevent the reconstruction of trees with 100,000 plant species today. Encouragingly, methodological advancement in this field is proceeding rapidly [39]–[43].

In summary, we propose that the continued role of public sequence databases in evolutionary analysis at comprehensive scales will depend critically on advancement in at least three areas: (1) the continued expansion of taxonomic and genetic coverage of these data, (2) ongoing efforts to understand the effects of the complex structure of large phylogenetic datasets, and (3) innovative solutions to the challenges posed by their analysis.

## Analysis

### Data collection and phylogeny inference

Data were gathered from GenBank [1] release 185 (accessed in May 2012), using the software PHLAWD [7], which uses an algorithm based on recursive profile alignment (alignment of multiple alignments to one another) to facilitate the alignment of nucleotide sequence data even at relatively deep phylogenetic levels. PHLAWD requires vetted guide sequences to ensure accurate identification of candidate sequences for alignment. High-quality guide sequences for many loci were supplied by Moore et al. [44] and Soltis et al. [22], which we combined with strict coverage and identity requirements to ensure the inclusion of homologous candidate sequences (Table S1, lines with coverage and identity set at 0.4). For the remaining alignments, we manually selected guide sequences from GenBank, and optimized search parameters to ensure homology and minimize noise. Explicit search terms were used to exclude non-homologous sequences for some loci (Table S1, lines with coverage and identity set to 0), while for others we used coverage/identity cutoffs as well as search terms.

In some cases, loci with uncertain homology were aligned separately for different clades. In these cases, search parameters in Table S1 may appear to indicate that some alignments are taxonomically nested subsets of others, e.g. trnG_intron_bryos defines its search clade as Streptophytina whereas trnG_intron_tracheophyta appears to use an identical search over Tracheophyta only, but because Tracheophyta is nested within Streptophytina, it would seem given this nesting that both these alignments would contain Tracheophyta sequences. In this case, however, and in other similar cases, other information such as genetic distance from guide sequences was used to exclude sequences that would otherwise have been represented twice from the the more inclusive alignment.

Each of these PHLAWD alignments was imported into to a SQLite database where sequences were linked on the basis of ncbi taxon id. Using the NCBI taxonomy, we extracted a synthetic concatenated alignment from this database, with each OTU in the alignment corresponding to an NCBI-recognize genus, and each partition corresponding one of the alignments generated with PHLAWD (i.e. each partition corresponded to a single locus). To populate the alignment, we used exemplar sequences that were chosen on the basis of unaligned length; the longest sequence available for any species in each genus was used to exemplify that genus for each locus. The Python scripts that were used to create the SQLite database and query it are available in the github repository http://github.com/chinchliff/autophy.

The final concatenated alignment file (with empty columns removed) used to create Figs. 1, 5, and 6 consisted of 13,093 tips representing genera, and 128 partitions representing loci. This alignment contained 148,143 total sites encoding 126,121 site patterns, 96.37% missing data, and was 1.9 GB in size. It is available in the Data Dryad repository, as are the metadata for all GenBank sequences used in this alignment, including GI numbers. Phylogenetic trees were inferred from this alignment in RAxML 7.3.0 [15], using the command line arguments:

raxmlHPC-PTHREADS-SSE3 -f d -m GTRCAT -p 12345 -q <partitionfile> -s <alignmentfile> -n <name> -j -D -T <numthreads>

The raw topology corresponding to the ML best tree found by RAxML is supplied in Newick format with branch lengths in the Dryad repository. Several extremely long tip branches (potentially representing erroneous phylogenetic chimeras) were manually pruned from this tree for display in Figs. 1, 5, and 6. The pruned topology was used in conjunction with time calibrations from [31] to generate the ultrametric chronogram supplied in the Data Dryad repository, using the program treePL [45].

We used Python scripts (all available in the Dryad repo) to extract sequence age data from GenBank and calculate the ages of the oldest exemplar sequences used to color the tree in Fig. 1. First, the script get_gi_dates_from_gbseq.py was used to extract the date of every land plant sequence from the GenBank release 185 flatfiles. Second, the script calc_age_of_oldest_exemplar_for_nodes.py was used to find the earliest added sequence that could be used to exemplify each tip in the Fig. 1 topology (i.e. the sequence was a sample from a the taxon represented by the tip), and for each internal branch, to identify the age of the oldest exemplar sequence for any of the leaves subtended by that branch.

### Assessing the impact of missing data

Partial decisiveness (the 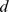 statistic as defined by Sanderson et al. [9]) for the entire dataset was estimated with the software Decisivator (J.W. Brown). We also calculated the number of loci with decisive taxon sampling for each branch (using the Python script calc_branchwise_decisiveness.py, available in the Dryad repo), which is simply a count of the data partitions from the alignment (i.e. loci) for which the four-way partition property of Steel and Sanderson [10] is satisfied for the given branch. The four-way partition property describes the distinguishability (or not) of an edge based on the presence of minimal data to inform that edge—this property is satisfied for an edge (i.e. the edge is distinguishable) if and only if the alignment contains sufficient taxon by locus sampling that it is *possible* for the given edge to be informed by at least some of the data. For a thorough mathematical exposition of the four-way partition property and its relevance for phylogenetic data decisiveness, we refer readers to the original publications [9], [10].

### Measuring branch support

We used a localized taxon quartet jackknife approach to subsample the original alignment in order to estimate support values. To explain this, we first define the rationale used for the taxonomic subsampling itself. Let 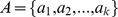 represent an alignment of phylogenetic data, where each 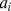 is a row in the alignment corresponding to a tip 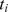 in a given rooted, bifurcating tree 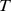 with tips 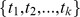 and internal edges 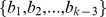. In such a tree, each observed internal edge 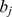 defines four non-overlapping subsets of 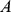: its daughter clades 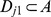 and 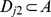, a sister clade 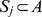, and the rest of the tree 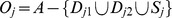. Then, for a given branch 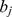, any bifurcating tree topology 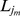 inferred for any taxon quartet 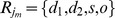 such that 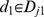, 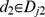, 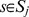, and 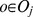, must either contain an internal edge representing the bipartition 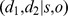, which is consistent with the tree topology containing 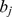, or else 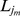 will contain an edge implying one of two alternative bipartitions that are inconsistent with 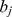 (these are 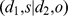 and 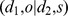). We therefore designate any taxon quartet 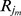 as a *representative taxon quartet replicate* for branch 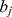, and 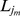 as a *representative quartet topology replicate*, for all 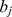 in 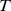.

We assessed support for each branch in the ML topology inferred using our large generic alignment, by performing 500 random taxon selection procedures to generate representative taxon quartet replicates, inferring the ML topology for each of these quartet replicates using data from the original alignment and RAxML [15], and then calculated the ICA score for the topology 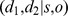 (consistent with the original ML tree), across all 500 of these representative quartet topology replicates for each branch. ICA is an information theory-based measure of edge support that is calculated across a set of topologies [33]. ICA varies on the interval 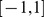, with negative values indicating that the targeted edge occurs less frequently across the replicate topologies than some other conflicting edge, positive values indicating that the edge occurs more frequently than any other conflicting edge, and a value of zero indicating that the edge occurs at equal frequency with all alternative (i.e. conflicting) edges. The absolute value of ICA is correlated with the overall frequency of that branch relative to alternative (i.e. conflicting) topologies in the input set. The calculations to yield these ICA scores were performed using software phyx [46].

Colored trees (Figs. 1, 5, and 6, and the “Supplemental tree figures” file in Dryad repository) were generated using the Python script paint_branches.py (available in the dryad repo) and the software FigTree. The paint_branches.py script cross-references node labels in a newick tree file with a CSV file containing values for those nodes, and then assigns branch colors based on those values. It generates a FigTree-formatted tree file with branch color annotations, which can be visualized in FigTree [47] itself and then exported in graphical formats. We used FigTree 1.4.0. The branch-painting script depends on the newick3.py and phylo3.py modules also provided in the Dryad repository.

### Additional procedures

Monophyly was assessed for all families in the GenBank taxonomy at the time of writing using the Python script test_monophyly_against_tree.py (available in the Dryad repo). The script accesses a taxonomy through a PHLAWD sequence database; we used a database containing the NCBI taxonomy from GenBank release 185. The test for monophyly involved two parts: first, for a given family, all the tips contained within that family in the taxonomy were identified in the tree. We note that in some cases, not all the taxa defined in the taxonomy were present in the tree. Second, the tree topology was checked to determine if the set of identified tips formed a monophyletic group in the tree, that is, they were all contained in a single clade that contained no other tips. If this condition was met, we inferred that monophyly could not be rejected for the given family. Conversely, if this condition was not met (i.e. the tips associated with a given family did not form a clade in the tree), then monopoly was rejected.

Figure 3 was created using the software Circos 0.62–1 [48]. The karyotype data used to assign genes and named regions were taken from the *Coffea arabica* chloroplast genome on GenBank (accession NC008535). The data for sampling frequencies for loci were extracted from the metadata files generated by the autophy scripts and formatted for Circos using a combination of bash and Python scripting and regular expression search/replace in the text editor Geany [49].

The estimate of time required to reach representation of 300,000 plant species in GenBank was based on an extrapolation using a linear rate estimate of species accumulation (about 6,500 new species/year) between 2000 and 2012. At this rate, it will take 32 years to accumulate the additional 208,000 species required to reach 300,000 from the approximately 92,000 sampled today.

## Supporting Information

Table S1 **A comma–separated tabular data file describing parameters for all PHLAWD runs.**
(CSV)

Table S2 **Proportion of branches in tree for which each number of loci contain decisive data.**
(CSV)

Table S3 **Number of genera represented for each locus in data mined from GenBank 185.**
(CSV)

File S1 **Full size version of** **Fig. 1** **with tip names.**
(PDF)

File S2 **Full size version of** **Fig. 5** **with tip names.**
(PDF)

File S3 **Full size version of** **Fig. 6** **with tip branches and tip names.**
(PDF)
