# Supplementary material for: Some Limitations of Public Sequence Data for Phylogenetic Inference (in Plants)
Source: PLoS One. 2014 Jul 7;9(7):e98986. doi: 10.1371/journal.pone.0098986 (PMC4085032; doi:10.1371/journal.pone.0098986)

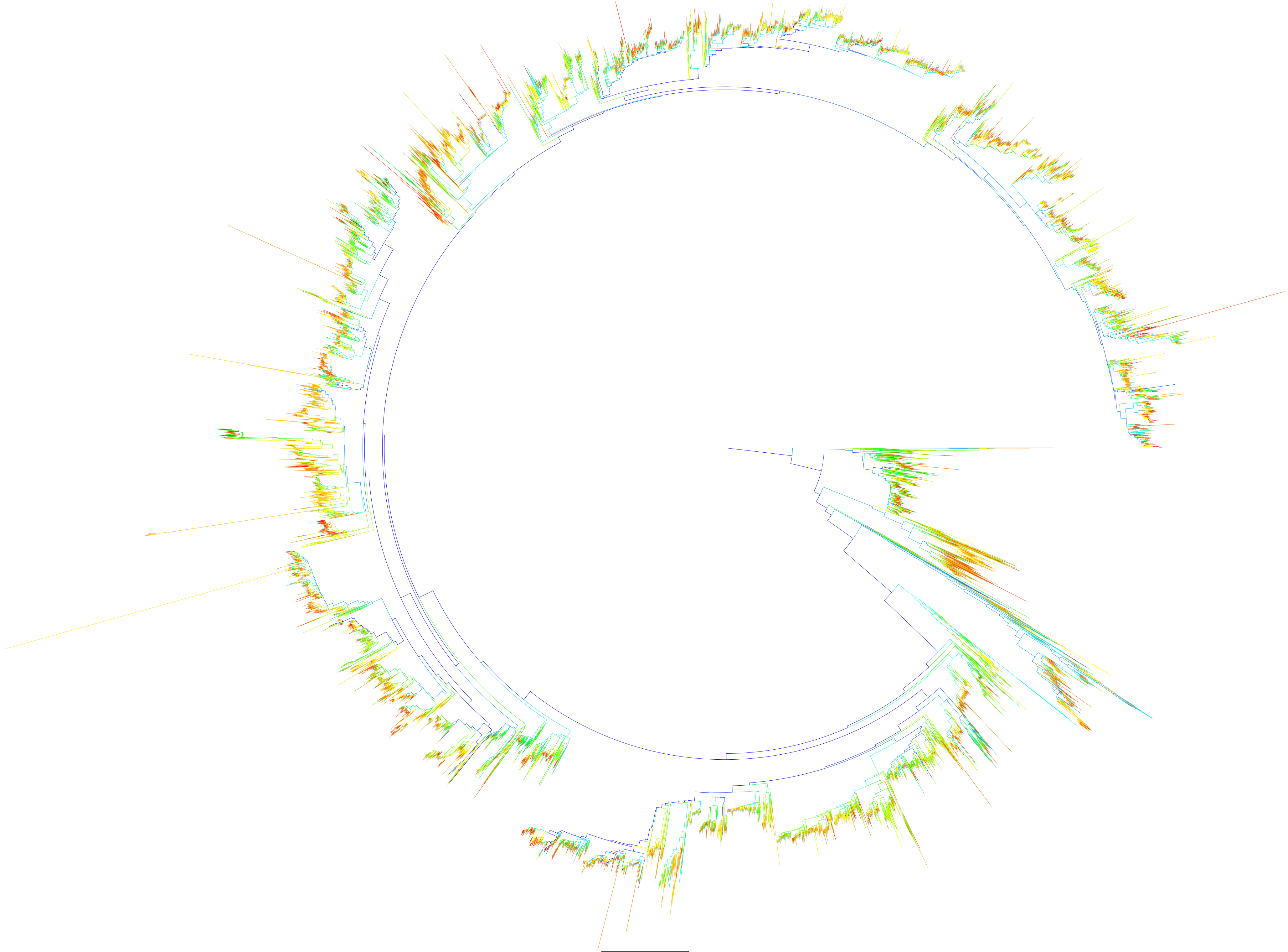

Supplement: File S1 — Full size version of Fig. 1 with tip names. (PDF) [file pone.0098986.s004.pdf]
